# Biofilm Formation in *Klebsiella pneumoniae* Bacteremia Strains Was Found to be Associated with CC23 and the Presence of *wcaG*

**DOI:** 10.3389/fcimb.2018.00021

**Published:** 2018-02-23

**Authors:** Jin-xin Zheng, Zhi-wei Lin, Chen Chen, Zhong Chen, Fo-jun Lin, Yang Wu, Si-yu Yang, Xiang Sun, Wei-ming Yao, Duo-yun Li, Zhi-jian Yu, Jia-lin Jin, Di Qu, Qi-wen Deng

**Affiliations:** ^1^Department of Infectious Diseases and the Key Laboratory of Endogenous Infection, Shenzhen Nanshan People's Hospital of Shenzhen University, Shenzhen, China; ^2^Quality Center of Hospital-Acquired Infection and Control, Shenzhen, China; ^3^Department of Infectious Diseases, Huashan Hospital, Fudan University, Shanghai, China; ^4^Key Laboratory of Medical Molecular Virology of Ministries of Education and Health, School of Basic Medical Science and Institutes of Biomedical Sciences, Shanghai Medical College of Fudan University, Shanghai, China

**Keywords:** *Klebsiella pneumonia*, bacteremia, biofilm formation, virulence genes, multilocus sequence typing

## Abstract

*Klebsiella pneumoniae* bacteremia biofilm traits and distribution characteristics have not been clarified. This study aimed to determine the prevalence and characteristics of *K. pneumoniae* bacteremia biofilm formation (BF) and to explore the virulence factors associated with *K. pneumoniae* BF. A total of 250 *K. pneumoniae* bacteremia isolates were collected from patients in Shenzhen and Shanghai, China. Virulence genes in their genomes were detected by PCR. The isolates were subjected to multilocus sequence typing (MLST) and clonal complex (CC) classification based on housekeeping genes. Biofilms were detected by crystal violet staining. Greater BF was observed in isolates from young adults (<40 years old) than in those from seniors (≥65 years old; *P* = 0.002). MLST yielded 65 different sequence types (STs), with the most represented STs being ST11, ST23, and ST65, and the main CCs were CC23 and CC65; CC23 isolates exhibited greater BF than CC65 or ST11 isolates (both *P* < 0.001). BF was more pronounced among *magA(K1), aero*+, *rmpA*+, *rmpA2*+, *allS*+, *wcaG*+, and *iutA*+ isolates than in isolates that were negative for these virulence factors. Multivariate regression analysis revealed only *wcaG* as an independent risk factor for BF (odds ratio 11.426, *P* < 0.001), and BF was decreased when *wcaG* was silenced by antisense RNA. In conclusion, BF in *K. pneumoniae* bacteremia isolates was found to be associated with CC23 classification and the presence of the *wcaG* virulence factor gene.

## Introduction

*Klebsiella pneumoniae* has been attracting increasing attention worldwide as an infectious microorganism due to the recent rise in the number of severe *K. pneumoniae* infections, antibiotic resistance, and growing difficulty with establishing effective treatments. *K. pneumoniae* is now the second most common cause of Gram-negative bacteremia and a major pathogen in hospital-acquired infection, particularly in immunocompromised patients (Candan and Aksöz, 2015; Paczosa and Mecsas, 2016). *K. pneumoniae* bacteremia has a high mortality rate (27.4–37.0%), especially when the strain is hypervirulent (Meatherall et al., 2009; Chetcuti-Zammit et al., 2014; Girometti et al., 2014; Li et al., 2014; Yu et al., 2016).

Relative to planktonic *K. pneumoniae* infections, infections with *K. pneumoniae* strains with the ability to form biofilms are more difficult to treat (Ribeiro et al., 2015). Diago-Navarro et al. (2014) found that nearly half of 40 examined carbapenem-resistant *K. pneumoniae* bacteremia strains were able to form obvious biofilms, including 13 that exhibited high levels of biofilm formation (BF). The antibiotic resistance of mature bacterial biofilm is 10–1,000 times that of planktonic bacteria, and bacteria in biofilms can resist phagocytosis, making them very challenging to eliminate (Lebeaux et al., 2014).

The phylogenetic relationships of bacterial pathogens can be described by multilocus sequence typing (MLST) and clonal complex (CC) classification (Urwin and Maiden, 2003). Virulence, drug resistance, and BF traits differ across bacterial isolates of different sequence types (STs) (Kozitskaya et al., 2005; Manning et al., 2009). ST27 of *Staphylococcus epidermidis* occurs preferentially in hospitals, and those ST27 strains with prevalent BF appear to adapt easily to nosocomial environments (Kozitskaya et al., 2005). Relative to other STs, the ST17 and ST19 lineages of group B *Streptococcus* strains isolated from invasive disease cases were found to be significantly more likely to form weak biofilms (Parker et al., 2016). ST23 isolates from carbapenem-resistant *K. pneumoniae* bacteremia samples were found to have higher BF than ST258 isolates (Diago-Navarro et al., 2014). However, the distributions of BF traits and STs among *K. pneumoniae*, especially in bacteremia, are still unknown in China.

In one study, type 1 and type 3 fimbriae were found to enhance *K. pneumoniae* BF on urinary catheters in a catheterized bladder model (Stahlhut et al., 2012). However, in another study, type 1 fimbriae were found not to influence BF, whereas expression of type 3 fimbriae was found to strongly promote BF and to favor the development of catheter-associated *K. pneumoniae* infections (Schroll et al., 2010). In a study examining pyogenic liver abscess *K. pneumoniae*, Wu et al. (2011) found that the genes *treC* and *sugE* affect BF by modulating capsular polysaccharide production and that *treC* facilitates gastrointestinal tract colonization, indicating that BF contributes to the establishment and persistence of *K. pneumoniae* infection.

The characteristics of *K. pneumoniae* that are associated with BF, including virulence factor expression, have not been clarified. Thus, the aim of the present study was to explore the prevalence and characteristics of *K. pneumoniae* bacteremia BF and to identify virulence factors associated with BF.

## Materials and methods

### Bacterial strains and clinical data collection

A total of 250 unique *K. pneumoniae* bacteremia isolates were collected from patients at Shenzhen Nanshan People's Hospital, Shenzhen University and Huashan Hospital, Fudan University in China between January 2010 and August 2017. The strains were identified with a Phoenix 100 automated microbiology system (BD, Franklin Lakes, NJ, USA), and then after two subcultured generations re-identified with matrix-assisted laser desorption ionization-time of flight mass spectrometry (IVD MALDI Biotyper, Bruker, Bremen, Germany). All procedures involving human participants were performed in accordance with the ethical standards of Shenzhen University and Fudan University, and the 1964 Helsinki declaration and its later amendments. For this type of study, formal consent is not required.

Clinical data were collected for each case from an electronic medical records database. Bacteremia origin was determined, based on bacteriological sampling at the suspected origin and clinical examination reports (Picot-Guéraud et al., 2015). In cases that were unclear, a second physician was consulted. We sought to determine the infection acquisition setting in each case. If this could not be confirmed and bacteremia occurred >48 h after hospital admission, hospital-acquired infection was presumed (Picot-Guéraud et al., 2015). If the bacteremia began <48 h after admission and at least one of Friedman's criteria (i.e., intravenous chemotherapy or hemodialysis in the last 30 days; home intravenous therapy or wound care in the last 30 days; hospitalization for ≥2 days in the last 90 days; or residence in a long-term care facility) was met, then the bacteremia was considered to be healthcare associated (Culshaw et al., 2014; Picot-Guéraud et al., 2015). In all other cases, the bacteremia was considered to be community acquired.

### Antibiotic susceptibility testing

The susceptibilities of the isolates to clinically relevant antibiotics (amikacin, cefotaxime, ceftazidime, cefepime, cefoperazone-sulbactam, chloramphenicol, ciprofloxacin, levofloxacin, gentamicin, piperacillin-tazobactam, tetracycline, imipenem, and meropenem) and extended-spectrum β-lactamase (ESBL) production were detected with a Phoenix 100 automated microbiology system (BD) (Saffert et al., 2011) or by the disk diffusion test (Chang et al., 2015). The minimal inhibitory concentrations (MICs) of imipenem, meropenem, tigecycline, and eravacycline were determined by the agar dilution method according to Clinical and Laboratory Standards Institute guidelines (Michail et al., 2013).

### String test

Strains with the hypermucoviscosity phenotype (as revealed by a positive string test result) were defined as hypervirulent *K. pneumoniae*; those with a negative result were defined as classic *K. pneumoniae*. A positive string test result was defined as the formation of a mucoviscous string of >5 mm on a bacteriology inoculation loop used to stretch a colony grown overnight on an agar plate at 37°C (Shon et al., 2013).

### MLST and CC

Bacterial DNA was extracted from isolates and purified with a DNeasy Blood and Tissue Kit (Qiagen China Co., Ltd, Shanghai, China) according to the manufacturer's protocol. MLST was conducted by the method of Diancourt et al. (2005). All primer sequences used in MLST are listed in Table S1. Briefly, seven housekeeping genes (*gapA, infB, mdh, pgi, phoE, rpoB*, and *tonB*) were PCR-amplified and sequenced from all isolates according to the *K. pneumoniae* MLST protocol (www.pasteur.fr/mlst). Alleles and sequence types (STs) were assigned by the MLST database (www.pasteur.fr/mlst/Kpneumoniae.html). *K. pneumoniae* CCs were identified by the eBURST v 3.0 program as described by Feil et al. (2004). CCs were defined as groups of two or more independent isolates that shared identical alleles at six or more loci; each complex was named after the putative founder ST (Wang et al., 2013).

### Biofilm assay

*K. pneumoniae* BF was detected by the method of Wu et al. (2011). Briefly, 1 μl of an overnight culture was inoculated into 100 μl of fresh Luria-Bertani (LB) broth in each well of a 96-well polystyrene plate (Costar3599, Corning, New York, USA). After 5 h of static incubation at 37°C, bacteria were stained with 25 μl of 0.5% crystal violet for 20 min. The supernatant was discarded, and plates were washed three times with deionized water to remove unattached cells. The biofilm-bound dye was then eluted with 95% ethanol, and the optical density at 550 nm (OD_550_) was determined. The NTUH-K2044 strain, which exhibits strong BF, served as a positive control (Wu et al., 2011). Each assay was performed in triplicate on at least three occasions.

### Detection of virulence genes by PCR

*K. pneumoniae* virulence genes were detected by PCR. The extracted genomic DNA from planktonic isolates served as a template for the amplification of virulence genes and for determining capsular serotypes. All PCR primer sequences and corresponding references are listed in Table S2. PCR amplification was performed in a total volume of 50 μl, containing 2 × PCR Master Mix (Tiangen Biotech Beijing Co., Ltd, Beijing, China), 0.5 mM of each primer, and 1 μl template DNA. The cycling conditions were as follows: 95°C for 3 min, followed by 30 cycles at 95°C for 30 s, 49°C−58°C for 30 s, 72°C for 60 s, and a final 10-min extension step at 72°C. Each PCR set included a no-template control and a positive control [NTUH-K2044 strain for *magA(K1)* and *wcaG*]. Amplification products were analyzed by electrophoresis in 1.0% agarose gels.

### Silencing of *wcaG* expression by antisense RNA

We used the antisense RNA gene silencing technique to construct *wcaG* antisense RNA silencing *K. pneumoniae* strains (Nakashima et al., 2006). The *wcaG* antisense RNA, which included the predicted Shine-Dalgarno sequence plus ~100 bp following the start codon, was amplified by PCR. PCR fragments were purified and digested with *Hin*dIII and *Bam*HI endonucleases and then inserted into the hairpin structure of plasmid pHN680 for gene silencing. Correct cloning was verified by PCR and sequencing. Verified loaded plasmids were introduced into three *K. pneumoniae* bacteremia isolates: Kp25, Kp57, and Kp63. All strains, plasmids, and primers used for RNA silencing are listed in Tables S3, S4. RNA silencing was induced with 1.0 mM isopropyl β-D-1-thiogalactopyranoside (IPTG). BF was detected by crystal violet as mentioned above. Growth of planktonic bacteria was measured with an OD_600_ assay. All assays were performed at least in triplicate.

### Quantitative real-time PCR

The expression levels of the *wcaG* were determined by quantitative real-time PCR (qRT-PCR). The primers used for qRT-PCR are listed in Table S4. Briefly, the total bacterial RNA was extracted by using an RNeasy Mini Kit (Qiagen China Co., Ltd, Shanghai, China). cDNA was then synthesized using a PrimeScript RT Reagent Kit (TaKaRa, Dalian, China). Finally, qRT-PCR was performed with a SYBR Premix Ex Taq II Kit (TaKaRa, Dalian, China) on the Mastercycler ep realplex System (Eppendorf, Hamburg, Germany), with an initial incubation at 95°C for 2 min, followed by 40 cycles of 15 s at 95°C and 60 s at 60°C. Each reaction was carried out in triplicate.

For all samples, a housekeeping gene (16S rRNA gene, *rrsE*) was used to normalize the expression of *wcaG*. The threshold cycle (CT) numbers were confirmed by the detection system software, and the data were analyzed based on the 2^−ΔΔ*Ct*^ method. The expression levels of *wcaG* were determined and compared with those of wildtype strains (expression = 1).

### Statistical analysis

The data, which are reported as means ± standard deviations (*SD*s), were analyzed with Student's *t*-test, one-way factorial analysis of variance, or the nonparametric Mann–Whitney *U*-test. Virulence factor association with BF was determined with a multivariate logistic regression model constructed based on the Wald statistic. Odds ratios (ORs) are reported with 95% confidence intervals (CIs). *P* < 0.05 were regarded as statistically significant. All data were analyzed in SPSS version 14.0 (Chicago, IL, USA).

## Results

### Clinical characteristics and BF of *K. pneumoniae* bacteremia strains

OD_550_ microplate readings of isolates after crystal violet staining ranged from 0.05 to 3.5. The median OD_550_ value for NTUH-K2044 (control strain) was 2.180, a value indicative of strong BF as expected (Wu et al., 2011). As reported in Table 1, the average OD_550_ value of the 250 examined *K. pneumoniae* bacteremia isolates was 1.23 ± 0.52. Biofilm-forming *K. pneumoniae* prevalence was similar between male and female patients and was unrelated to various coexisting conditions, but was greater among young adults under 40 years old than among seniors over 65 years old (*P* = 0.002, Table 1). Biofilm-forming *K. pneumoniae* prevalence was unrelated to infection acquisition source (community, hospital, intensive care unit, medical unit, or surgical unit), but was more common among isolates from bacteremia cases that persisted beyond 72 h despite treatment than from bacteremia cases that were cleared within 72 h of commencing treatment (*P* < 0.001, Table 1).

**Table 1:** Clinical characteristics of *K. 1pneumoniae* bacteremia strains.

**Characteristics**	**No. isolates**	**OD_550_ (mean ± *SD*)**	***P*-value**
Total no. isolates tested	250	1.23 ± 0.52	
**Patient characteristics**			
Male	155	1.24 ± 0.52	0.765
Female	95	1.22 ± 0.51	
Age < 40 years	49	1.42± 0.69	**0.002**^a^
40 years ≤ age ≤ 65 years	120	1.26 ± 0.47	
65 years < age	81	1.07 ± 0.40	
**Comorbidity**			0.125
Pulmonary disease	21	1.21 ± 0.53	
Cancer	43	1.06 ± 0.36	
Heart disease	48	1.25 ± 0.45	
Stroke	17	1.13 ± 0.28	
Kidney disease	20	1.25 ± 0.46	
Diabetes mellitus	58	1.17 ± 0.39	
Liver disease	34	1.03 ± 0.32	
Immunosuppression	24	1.06 ± 0.41	
**Acquisition**			
Community acquired	115	1.17 ± 0.43	0.062
Hospital acquired	135	1.29 ± 0.58	
Intensive care unit	44	1.12 ± 0.41	0.233
Medical unit	124	1.22 ± 0.51	
Surgical unit	53	1.33 ± 0.56	
Other	29	1.28 ± 0.59	
**Bacteremia source**			0.558
Catheter-related infection	8	1.26 ± 0.30	
Respiratory tract infection	99	1.17 ± 0.44	
Intra-abdominal infection	54	1.24 ± 0.53	
Urinary tract infection	27	1.28 ± 0.44	
Unknown or primary bacteremia	62	1.30 ± 0.65	
**Bacteremia outcome**			
Clearance after 72-h treatment	51	0.92 ± 0.21	<**0.001**
Persistence after 72-h treatment	165	1.33 ± 0.50	

a *Age < 40 years vs. age > 65 years. All the bold values with significant difference between those groups (All p < 0.05)*.

### BF and antibiotic susceptibility

BF prevalence did not differ between antibiotic-resistant and -susceptible *K. pneumoniae* isolates for any of the tested antibiotic drugs (Table 2). MIC levels (MIC < 1 mg/L, 1 ≤ MIC mg/L < 4 or MIC ≥ 4 mg/L) for imipenem, meropenem, tigecycline, and eravacycline were not significantly related to BF among *K. pneumoniae* isolates (Table 2). The capacity for ESBL production was also unrelated to BF.

**Table 2:** Analysis of BF and antibiotic resistance in *K. pneumoniae* isolates.

**Antibiotic**	**Resistant/sensitive or MIC level (mg/L)**	**No. isolates**	**OD_550_ (mean ± *SD*)**	***P*-value**
Cefotaxime	Resistance	83	1.16 ± 0.45	0.124
	Sensitive	161	1.26 ± 0.54	
Ceftazidime	Resistance	61	1.17 ± 0.46	0.243
	Sensitive	181	1.26 ± 0.54	
Cefepime	Resistance	71	1.16 ± 0.44	0.155
	Sensitive	171	1.25 ± 0.53	
Cefoperazone-sulbactam	Resistance	51	1.23 ± 0.50	0.914
	Sensitive	186	1.24 ± 0.52	
Piperacillin-tazobactam	Resistance	59	1.21 ± 0.49	0.513
	Sensitive	175	1.27 ± 0.53	
Chloramphenicol	Resistance	64	1.16 ± 0.39	0.152
	Sensitive	177	1.26 ± 0.55	
Gentamicin	Resistance	65	1.18 ± 0.39	0.258
	Sensitive	184	1.25 ± 0.26	
Ciprofloxacin	Resistance	65	1.17 ± 0.41	0.146
	Sensitive	175	1.26 ± 0.56	
Levofloxacin	Resistance	60	1.16 ± 0.42	0.173
	Sensitive	183	1.25 ± 0.55	
Tetracycline	Resistance	93	1.21 ± 0.46	0.716
	Sensitive	151	1.24 ± 0.55	
ESBL production	Yes	70	1.17 ± 0.43	0.222
	No	180	1.25 ± 0.55	
Imipenem	MIC < 1	190	1.26 ± 0.56	0.101
	1 ≤ MIC < 4	21	1.23 ± 0.37	
	MIC ≥ 4	39	1.07 ± 0.31	
Meropenem	MIC < 1	177	1.27 ± 0.57	0.110
	1 ≤ MIC < 4	19	1.17 ± 0.42	
	MIC ≥ 4	54	1.11 ± 0.29	
Tigecycline	MIC < 1	72	1.20 ± 0.38	0.721
	1 ≤ MIC < 4	145	1.25 ± 0.27	
	MIC ≥ 4	33	1.19 ± 0.56	
Eravacycline	MIC < 1	164	1.27 ± 0.56	0.123
	1 ≤ MIC < 4	74	1.18 ± 0.45	
	MIC ≥ 4	12	0.99 ± 0.20	

### BF and the MLST of *K. pneumoniae* isolates

**Figure 1:**
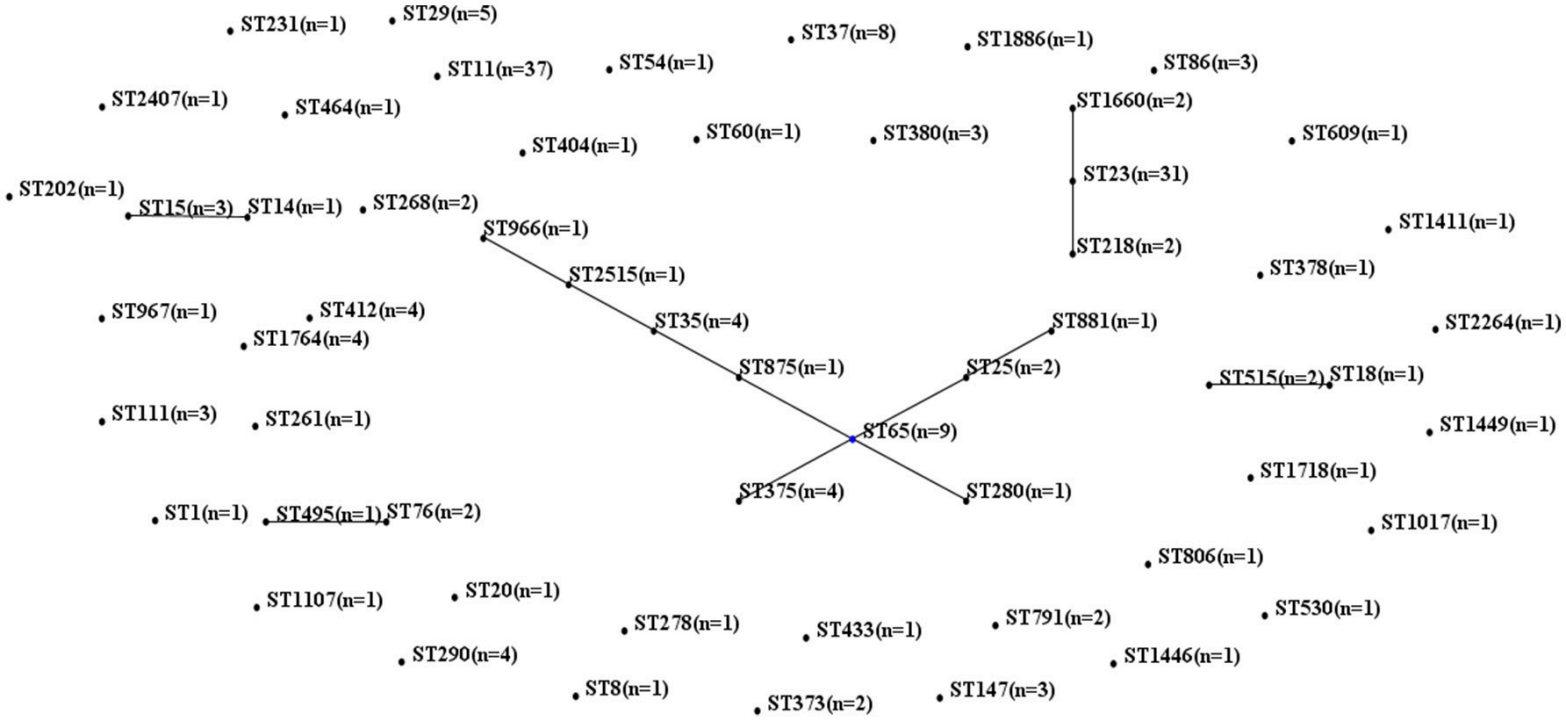
Characteristics of MLST and CC analysis of 187 *K. pneumoniae* isolates. Lines connecting STs indicate CCs, including CC23 (vertical line) and CC65 (crossing lines).

**Figure 2:**
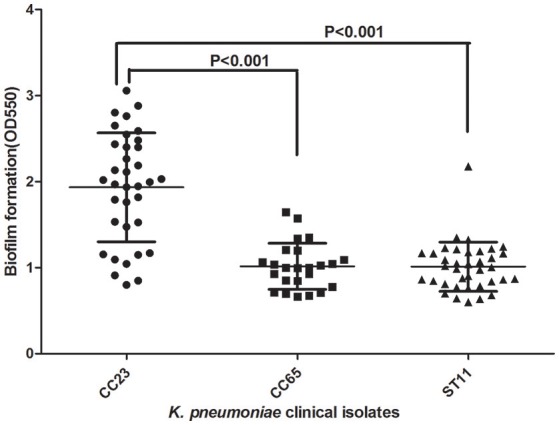
Comparison of BF between CC23, CC65, and ST11 isolates. A Student's *t*-test indicated significantly greater BF among the CC23 isolates than among the CC65 or ST11 isolates. Error bars show *SD*.

MLST of the 250 *K. pneumoniae* bacteremia strains yielded clear results for 187 isolates, including 65 different STs. The biofilm characteristics and numbers of isolates assigned to each ST are shown in Table S5. Because the STs widely variation among these *K. pneumoniae* isolates, CCs were analyzed based on STs in eBURST (Figure 1). The main CCs of the *K. pneumoniae* bacteremia isolates were CC23 and CC65. This study also identified ST11 as the main ST among the *K. pneumoniae* bacteremia isolates. Thus, further analysis of the BF characteristics of CC23, CC65, and ST11 isolates (Figure 2) indicated that BF tended to be stronger among CC23 isolates than among CC65 isolates (*P* < 0.001) or ST11 isolates (*P* < 0.001).

### Correlation between BF and virulence factors

As reported in Table 3, analysis of PCR-amplified virulence factors showed that BF was more pronounced among *magA(K1)*-positive (+) isolates than among *magA(K1)*-negative (–) isolates. Additionally, BF was more pronounced among *aero*+, *rmpA*+, *rmpA2*+, *allS*+, *wcaG*+, and *iutA*+ isolates than among isolates that were negative (–) for these virulence factors. However, BF was not increased in hypermucoviscous or *K2A(K2)* serotype positive (+) isolates compared with isolates not expressing each of the two virulence factors.

**Table 3:** Relationship between BF capacity and virulence factors in *K. pneumoniae* isolates.

**Virulence factor**	**No. isolates**	**OD_550_ (mean ± *SD*)**	***P*-value**
Hypermucoviscosity+	58	1.26 ± 0.62	0.741
Hypermucoviscosity-	192	1.23 ± 0.48	
*magA(K1)+*	43	1.82 ± 0.68	<**0.001**
*magA(K1)–*	207	1.11 ± 0.38	
*K2A(K2)+*	27	0.99 ± 0.26	<**0.001**
*K2A(K2)–*	223	1.26 ± 0.53	
*aero+*	94	1.44 ± 0.66	<**0.001**
*aero–*	156	1.11 ± 0.36	
*rmpA+*	119	1.37 ± 0.63	<**0.001**
*rmpA–*	131	1.11 ± 0.35	
*rmpA2+*	81	1.39 ± 0.67	**0.006**
*rmpA2–*	169	1.16 ± 0.41	
*allS+*	55	1.57 ± 0.70	<**0.001**
*allS–*	195	1.14 ± 0.41	
*wcaG+*	47	1.87 ± 0.65	<**0.001**
*wcaG–*	203	1.08 ± 0.35	
*wabG+*	197	1.24 ± 0.55	0.410
*wabG–*	53	1.19 ± 0.37	
*fimH+*	183	1.26 ± 0.55	0.102
*fimH–*	67	1.16 ± 0.40	
*mrkD+*	70	1.28 ± 0.34	0.226
*mrkD–*	180	1.21 ± 0.57	
*iutA+*	101	1.35 ± 0.65	**0.009**
*iutA–*	149	1.16 ± 0.39	
*cnf+*	55	1.26 ± 0.38	0.671
*cnf–*	195	1.23 ± 0.55	

*+, positive; –, negative. All the bold values with significant difference between those groups (All p < 0.05)*.

### Virulence factor association with BF: multiple regression analysis

In order to determine the independent contribution of each virulence factor to the *K. pneumoniae* bacteremia BF, multiple logistic regression analysis was conducted. As shown in Table 4, of eight independent variables [*magA(K1), K2A(K2), wcaG, aero, rmpA, rmpA2, alls*, and *iutA*] submitted to a logistic regression model constructed by a backward selection approach based on the Wald statistic (dependent variable: OD_550_-value ≥ 1.25), only one factor, *wcaG*, was found to be an independent risk factor for BF of *K. pneumoniae* bacteremia isolates (OR 11.426, *P* < 0.001). Notably, *magA(K1)* was not associated with the BF of *K. pneumoniae* bacteremia isolates.

**Table 4:** Multivariate regression analysis of virulence factor association with *K. pneumoniae* BF.

**Factor**	**OR (95% CI)**	***P*-value**
*wcaG*	11.426 (4.989–26.171)	<**0.001**
*aero*	1.847 (0.893–3.819)	0.098
*rmpA2*	0.485 (0.223–1.058)	0.069

*OR, odds ratio; CI, confidence interval. The bold value with significant difference between those groups (p < 0.05)*.

### *wcaG* associated with BF was detected by RNA silencing

**Figure 3:**
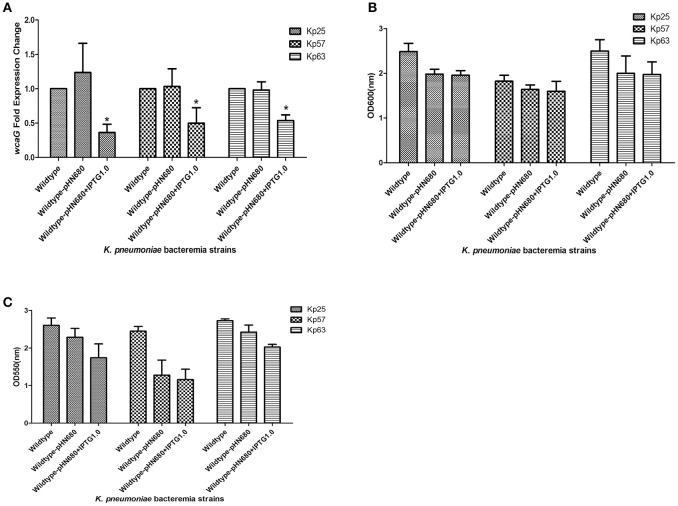
Silencing of *wcaG* expression by antisense RNA. Expression levels of *wcaG* were determined by qRT-PCR; ^*^wildtype-pHN680+IPTG vs. wildtype-pHN680, *P* < 0.05 (Student's *t*-test) **(A)**. Growth of planktonic bacteria was measured by optical density at 600 nm **(B)**. Biofilm formation of *K. pneumoniae* bacteremia strains was detected by crystal violet and the optical density determined at 550 nm **(C)**. Errors bars represent mean ± *SD* or three replicates.

In order to confirm the role of *wcaG* in the BF of *K. pneumoniae* bacteremia, *wcaG* was silenced by antisense RNA. As Figure 3A indicates, the expression levels of *wcaG* decreased by half following IPTG induction. RNA silencing of *wcaG* expression with pHN680 reduced growth, as indexed by OD_600_, of Kp25, Kp27, and Kp63 isolates relative to wildtype (no plasmid) controls, and the inducer IPTG (1.0 mM) had no apparent effect on the growth of the three pHN680-containing isolates (Figure 3B). Interestingly, the BF, as indexed by OD_550_, of Kp25 and Kp63 strains was decreased following IPTG induction, relative to controls not exposed to IPTG (Figure 3C).

## Discussion

In the present study, we found that BF was greater among isolates from young adults than among isolates from seniors. This differs somewhat from a previous study of sputum samples that found greater BF among isolates originating from patients over 70 years old than in those from patients under 70 years old (Yang and Zhang, 2008). This difference could be related to patients under 40 years old having stronger immune systems that put pressure on the bacteria to form biofilms in order to evade host immunity (Gunn et al., 2016). Our finding that BF was associated with persistent bacteremia infection after 72 h of treatment affirms that the bacteria in biofilms were very difficult to eliminate.

The relationship between *K. pneumoniae* BF and antibiotic resistance is uncertain. In the present study, we did not observe a significant association between BF and resistance to any one of the panel of antibiotics examined, nor between BF and ESBL production. However, Yang and Zhang (2008) reported that among 137 *K. pneumoniae* strains from sputum and urine, 85.0% (51/60) of biofilm-positive strains had the ability to produce ESBLs, while the rate was only 11.7% (9/77) for biofilm-negative strains, with the ESBL production rates being similar for isolates from blood and wound samples. Meanwhile, also differing from the present results, in a study of 100 urine samples, Subramanian et al. (2012) found 83.3 and 73.3% resistance rates to ampicillin and cefotaxime, respectively, among biofilm-forming isolates compared with only 60 and 35% resistance rates, respectively, among non-biofilm-forming isolates. Thus, the relationship between *K. pneumoniae* BF and antibiotic resistance remains controversial and needs to be further explored.

*K. pneumoniae* BF traits may vary among different geographical regions and STs. We observed a wide diversity of *K. pneumoniae* STs in our sample located in eastern and southern China. The most represented STs were ST11 (*n* = 37), ST23 (*n* = 31), and ST65 (*n* = 9), and the main CCs were CC23 and CC65. Prior research has shown a predominance of ST23 in east China (Wang et al., 2013; Qu et al., 2015). The ST11 type, as the most prevalent carbapenem-resistant *K. pneumoniae* type in China, was also recently found associated with hypervirulent or hypermucoviscous isolates of *K. pneumonia* (Bi et al., 2017; Zhan et al., 2017; Gu et al., 2018). However, the traits of BF among CC23, CC65, and ST11 are still unknown in China. Previously, BF ability has been reported to be higher for ST23 isolates than for ST258 isolates from carbapenem-resistant *K. pneumoniae* bacteremia samples (Diago-Navarro et al., 2014). The present study found that the CC23 isolates had a higher BF rate than the CC65 or ST11 isolates in China. Thus, those CC23 *K. pneumoniae* bacteremia isolates may be like *S. epidermidis* ST27 in terms of occurring preferentially in nosocomial environments and forming biofilms to better survive in adverse environmental conditions, including the presence of antibiotics and disinfectants (Kozitskaya et al., 2005; Lebeaux et al., 2014).

Currently, the role of virulence factors, such as *magA(K1), K2A(K2)*, and hypermucoviscosity, in the BF of *K. pneumoniae* bacteremia remains poorly understood. The present study found that *magA(K1)*- or *wcaG*-positive isolates exhibited a greater BF ability than hypermucoviscous- or *K2A(K2)*-positive isolates. This is similar to previous research, which found that hypermucoviscosity was not associated with *K. pneumoniae* BF (Soto et al., 2017). Although BF was more pronounced in isolates that were positive for virulence factors, such as *magA(K1), aero, rmpA*, and *rmpA2*, than in isolates that were negative for those virulence factors, the present study found that only *wcaG* played an important role in *K. pneumoniae* bacteremia BF. The *wcaG*-positive genotype has been associated with the K1 and K54 capsular types, as well as with, to a lesser extent, the K16 and K58 capsular types (Turton et al., 2010). The isolation of K1 and K54 *wcaG*-positive strains from hospital origins and from patients with invasive and serious infections indicates that *wcaG* may contribute to the virulence of these strains. Indeed, the protein encoded by *wcaG* participates in the biosynthesis of fucose (Ho et al., 2011), whose inclusion as a component of the polysaccharide capsule of *K. pneumoniae* has been associated with bacterial virulence in mice (Wu et al., 2008). Interestingly, our study is the first to our knowledge to identify that *wcaG* is an important factor in *K. pneumoniae* bacteremia BF. However, the mechanism by which *wcaG* facilitates *K. pneumoniae* BF has not been elucidated. Prior work showing that *wcaG* deletion mutations affect a large portion (15/20) of capsule polysaccharide genes (Ho et al., 2011) suggests that *wcaG* may promote *K. pneumoniae* BF by altering the composition of the microbe's polysaccharide capsule.

In conclusion, the present study showed that many *K. pneumoniae* bacteremia strains form biofilms readily. BF tended to be greater in isolates from young adults than in those from seniors. MLST demonstrated substantial *K. pneumoniae* ST diversity in our sample group; the most represented STs were ST11, ST23, and ST65, and the main CCs were CC23 and CC65. BF was more strongly associated with CC23 isolates than with CC65 or ST11 isolates. We found that only *wcaG* was associated with *K. pneumoniae* bacteremia BF. This study may help to elucidate the epidemic characteristics of *K. pneumoniae* bacteremia biofilms and how biofilm traits are associated with virulence factors in *K. pneumoniae* bacteremia worldwide.

## Author contributions

JZ: participated in the design of the study, carried out the biofilm assay and RNA silencing test, analyzed, and interpreted the data, and drafted the manuscript. ZL and ZC: performed antibiotic susceptibility testing, detected virulence genes by PCR, carried out the RNA silencing test, and participated in the data analysis. FL and YW: conducted the MLST and CC analysis, and biofilm assay, and provided a critical revision of the manuscript. XS,WY, and DL: participated in the acquisition of the samples, isolated DNA, conducted MLST and biofilm assay, and participated in the data analysis. ZY, DQ, and QD: designed the study, participated in the data analysis, and provided critical revisions of themanuscript for important intellectual content. CC: performed antibiotic susceptibility testing, detected virulence genes by PCR and participated in the data analysis. SY: conducted the MLST and CC analysis, and biofilm assay. JJ: participated in the data analysis, and provided critical revisions of the manuscript for important intellectual content.

### Conflict of interest statement

The authors declare that the research was conducted in the absence of any commercial or financial relationships that could be construed as a potential conflict of interest.

## Abbreviations

BF: biofilm formation
CI: confidence interval
CC: clonal complex
ESBL: extended spectrum β-lactamase
IPTG: isopropyl β-D-1-thiogalactopyranoside
MIC: minimal inhibitory concentration
MLST: multilocus sequence typing
OD: optical density
OR: odds ratio
PCR: polymerase chain reaction
SD: standard deviation
ST: sequence type.
