# Supplementary material for: Biofilm Formation in Klebsiella pneumoniae Bacteremia Strains Was Found to be Associated with CC23 and the Presence of wcaG
Source: Front Cell Infect Microbiol. 2018 Feb 23;8:21. doi: 10.3389/fcimb.2018.00021 (PMC5829044; doi:10.3389/fcimb.2018.00021)
Supplement: Supplementary file 1 [file Table1.DOC]

**Table S1. PCR primers used for *K. pneumoniae* MLST gene diversity determination.**

| **Target** | **Primer** | **Primer sequence (5'-3')** | **Amplicon size (bp)** | **Source** |
| --- | --- | --- | --- | --- |
| *gapA* | *gapA*-F | TGAAATATGACTCCACTCACGG | 662 | Diancourt et al., 2005 |
|  | *gapA*-R | CTTCAGAAGCGGCTTTGATGGCTT |  |  |
| *infB* | *infB*-F | CTCGCTGCTGGACTATATTCG | 462 | Diancourt et al., 2005 |
|  | *infB*-R | CTCGCTGCTGGACTATATTCG |  |  |
| *mdh* | *mdh*-F | CCCAACTCGCTTCAGGTTCAG | 756 | Diancourt et al., 2005 |
|  | *mdh*-R | CCGTTTTTCCCCAGCAGCAG |  |  |
| *pgi* | *pgi*-F | GAGAAAAACCTGCCTGTACTGCTGGC | 566 | Diancourt et al., 2005 |
|  | *pgi*-R | CGCGCCACGCTTTATAGCGGTTAAT |  |  |
| *phoE* | *phoE*-F | ACCTACCGCAACACCGACTTCTTCGG | 602 | Diancourt et al., 2005 |
|  | *phoE*-R | TGATCAGAACTGGTAGGTGAT |  |  |
| *rpoB* | *rpoB* **-F** | GGCGAAATGGCWGAGAACCA | 1075 | Diancourt et al., 2005 |
|  | *rpoB* **-R** | GAGTCTTCGAAGTTGTAACC |  |  |
| *tonB* | *tonB*-F | CTTTATACCTCGGTACATCAGGTT | 539 | Diancourt et al., 2005 |
|  | *tonB*-R | ATTCGCCGGCTGRGCRGAGAG |  |  |

F, forward; R reverse.
