# Supplementary material for: Biofilm Formation in Klebsiella pneumoniae Bacteremia Strains Was Found to be Associated with CC23 and the Presence of wcaG
Source: Front Cell Infect Microbiol. 2018 Feb 23;8:21. doi: 10.3389/fcimb.2018.00021 (PMC5829044; doi:10.3389/fcimb.2018.00021)
Supplement: Supplementary file 2 [file Table2.DOC]

**Table S2. PCR primers used for detection of *K.pneumoniae* virulence factors and capsular serotypes**.

| **Target** | **Primer** | **Primer sequence (5'-3')** | **Amplicon size (bp)** | **Source** |
| --- | --- | --- | --- | --- |
| *aero* | *aero*-F | GCATAGGCGGATACGAACAT | 556 | Candan and Aksöz, 2015 |
|  | *aero*-R | CACAGGGCAATTGCTTACCT |  |  |
| ***magA*** | ***magA*-F** | GGTGCTCTTTACATCATTGC | 1283 | Li et al., 2014 |
|  | *magA*-R | GCAATGGCCATTTGCGTTAG |  |  |
| *K2A* | *K2A -F* | GACCCGATATTCATACTTGACAGAG | 641 | Li et al., 2014 |
|  | *K2A -R* | CCTGAAGTAAAATCGTAAATAGATGGC |  |  |
| *rmpA* | *rmpA*-F | ACTGGGCTACCTCTGCTTC | 516 | Yu et al., 2016 |
|  | *rmpA*-R | CTTGCATGAGCCATCTTTCA |  |  |
| *rmpA2* | *rmpA2*-F | TGTGCAATAAGGATGTTACATTAGT | 535 | Yu et al., 2016 |
|  | *rmpA2*-R | TTTGATGTGCACCATTTTTCA |  |  |
| *allS* | *allS*-F | CCGAAACATTACGCACCTTT | 508 | Candan and Aksöz, 2015 |
|  | *allS*-R | ATCACGAAGAGCCAGGTCAC |  |  |
| *wcaG* | *wcaG*-F | GGTTGGKTCAGCAATCGTA | 169 | Candan and Aksöz, 2015 |
|  | *wcaG*-R | ACTATTCCGCCAACTTTTGC |  |  |
| *wabg* | *wabg*-F | ACCATCGGCCATTTGATAGA | 683 | Candan and Aksöz, 2015 |
|  | *wabg*-R | CGGACTGGCAGATCCATATC |  |  |
| *fimH* | *fimH*-F | TGCTGCTGGGCTGGTCGATG | 688 | Candan and Aksöz, 2015 |
|  | *fimH*-R | GGGAGGGTGACGGTGACATC |  |  |
| *mrkD* | *mrkD*-F | TTCTGCACAGCGGTCCC | 240 | Candan and Aksöz, 2015 |
|  | *mrkD*-R | GATACCCGGCGTTTTCGTTAC |  |  |
| *iutA* | *iutA*-F | GGCTGGACATCATGGGAACTGG | 300 | Candan and Aksöz, 2015 |
|  | *iutA*-R | CGTCGGGAACGGGTAGAATCG |  |  |
| *cnf* | *cnf*-F | AAGATGGAGTTTCCTATGCAGGAG | 498 | Candan and Aksöz, 2015 |
|  | *cnf*-R | CATTCAGAGTCCTGCCCTCATTATT |  |  |

F, forward; R reverse.
