# Supplementary material for: Biofilm Formation in Klebsiella pneumoniae Bacteremia Strains Was Found to be Associated with CC23 and the Presence of wcaG
Source: Front Cell Infect Microbiol. 2018 Feb 23;8:21. doi: 10.3389/fcimb.2018.00021 (PMC5829044; doi:10.3389/fcimb.2018.00021)
Supplement: Supplementary file 3 [file Table3.DOC]

**Table S3. Strains and plasmids used for the *K. pneumoniae wcaG* RNA silencing.**

| **Strain or plasmid** | **Relevant antibiotic resistance^a^** | **Description** | **Source** |
| --- | --- | --- | --- |
| *K. pneumoniae strain* | |  |  |
| Kp25 | Cm^50^, Erm^50^, Tet^100^, Amp^100^ | Bacteremia isolate, strong biofilm producer | This study |
| Kp57 | Cm^50^, Erm^50^, Tet^100^, Amp^100^ | Bacteremia isolate, strong biofilm producer | This study |
| Kp63 | Cm^50^, Erm^50^, Tet^100^, Amp^100^ | Bacteremia isolate, strong biofilm producer | This study |
| *Plasmid* |  |  |  |
| pHN678 | Cm^34^ | Shuttle vector with hairpin structure for gene silencing | Nakashima et al., 2006 |
| pHN679 | Cm^34^, Kan^50^ | pHN678 containing a 1.2-kb kanamycin resistance fragment | This study |
| pHN680 | Cm^34^, Kan^50^ | pHN679 containing a 130bp *wcaG*-antisense RNAs fragment | This study |

*^a^* Superscripts indicate the concentrations of antibiotics (in µg/ml) to which the strains and plasmids are resistant; Cm, chloramphenicol; Erm, erythromycin; Kan, kanamycin; Tet, tetracycline; Amp, ampicillin.
