# Supplementary material for: Biofilm Formation in Klebsiella pneumoniae Bacteremia Strains Was Found to be Associated with CC23 and the Presence of wcaG
Source: Front Cell Infect Microbiol. 2018 Feb 23;8:21. doi: 10.3389/fcimb.2018.00021 (PMC5829044; doi:10.3389/fcimb.2018.00021)
Supplement: Supplementary file 4 [file Table4.DOC]

**Table S4. PCR primers used for *K. pneumoniae wcaG* RNA silencing.**

| **Primers** | **Sequence (5' →3' )** | **Use** | **Source or reference** |
| --- | --- | --- | --- |
| Kan-F | CTAGCTAGCGAGTCCAACCCGGTAAGACAC | Amplification of kanamycin resistance fragment | This study |
| Kan-R | AAAAGTACTTTAGAAAAACTCATCGAGCAT | Amplification of kanamycin resistance fragment | This study |
| ASwacG-F | CCCAAGCTTCAATTTCTCTGGAGTAAAGAA | Amplification of wcaG-antisense RNAs fragment | This study |
| ASwcaG-R | CGCGGATCCAGCTCCTGACGTGAACGCAGT | Amplification of wcaG-antisense RNAs fragment | This study |
| JAswcaG-F | GCATACTCTGCGACATCGTAT | Screening of pHN680-wcaG antisense RNAs | This study |
| JASWcaG-R | TGGTTCAGCAATCGTAAGACA | Screening of pHN680-wcaG antisense RNAs | This study |
| rrsE-F | CTACAATGGCATATACAA | housekeeping gene for qRT-PCR | This study |
| rrsE-R | TTCTGATCTACGATTACT | housekeeping gene for qRT-PCR |  |
| RwcaG-F | CGATAAGCGAGTCTGAAT | qRT-PCR for determining the expression levels of *wcaG* | This study |
| RwcaG-R | TCTCTATTATGTTGCCGATT | qRT-PCR for determining the expression levels of *wcaG* |  |

F, forward; R reverse.
