# Supplementary material for: Biofilm Formation in Klebsiella pneumoniae Bacteremia Strains Was Found to be Associated with CC23 and the Presence of wcaG
Source: Front Cell Infect Microbiol. 2018 Feb 23;8:21. doi: 10.3389/fcimb.2018.00021 (PMC5829044; doi:10.3389/fcimb.2018.00021)
Supplement: Supplementary file 5 [file Table5.DOC]

**Table S5. BF by ST of 187 *K. pneumoniae* isolates.**

| **ST** | **OD550 (mean ± SD)** | **ST** | **OD550 (mean ± SD)** |
| --- | --- | --- | --- |
| 1 (n = 1) | 0.651 | 412 (n = 4) | 0.91 ± 0.34 |
| 8 (n = 1) | 0.911 | 433 (n = 1) | 1.327 |
| 11 (n = 37) | 1.01 ± 0.29 | 464 (n = 1) | 1.135 |
| 14 (n = 1) | 1.099 | 495 (n = 1) | 1.583 |
| 15 (n = 3) | 1.00 ± 0.19 | 515 (n = 2) | 1.14 ± 0.01 |
| 18 (n = 1) | 1.547 | 530 (n = 1) | 0.921 |
| 20 (n = 1) | 1.794 | 609 (n = 1) | 1.481 |
| 23 (n = 31) | 2.04 ± 0.58 | 632 (n = 1) | 1.013 |
| 25 (n = 2) | 0.74 ± 0.06 | 700 (n = 3) | 1.82 ± 0.19 |
| 29 (n = 5) | 1.21 ± 0.45 | 791 (n = 2) | 1.19 ± 0.33 |
| 35 (n = 4) | 1.04 ± 0.23 | 806(n = 1) | 1.269 |
| 37 (n = 8) | 1.12 ± 0.25 | 875 (n = 1) | 0.713 |
| 54 (n = 1) | 0.856 | 881 (n = 1) | 1.065 |
| 60 (n = 1) | 1.671 | 966 (n = 1) | 1.046 |
| 65 (n = 9) | 1.07± 0.26 | 967 (n = 1) | 1.064 |
| 76 (n = 2) | 1.20 ± 0.53 | 1017 (n = 1) | 1.198 |
| 86 (n = 3) | 0.78 ± 0.13 | 1049 (n = 2) | 1.25 ± 0.15 |
| 111 (n = 3) | 0.91 ± 0.15 | 1107 (n = 1) | 1.009 |
| 133 (n = 1) | 0.680 | 1333 (n = 1) | 2.245 |
| 147 (n = 3) | 1.11 ± 0.31 | 1411 (n = 1) | 1.09 |
| 202 (n = 1) | 0.985 | 1446 (n = 1) | 0.726 |
| 218 (n = 2) | 0.88 ± 0.04 | 1449 (n = 1) | 0.770 |
| 231 (n = 1) | 1.025 | 1469 (n = 1) | 0.856 |
| 261 (n = 1) | 1.491 | 1584 (n = 1) | 0.82 |
| 268 (n = 2) | 0.79 ± 0.25 | 1660 (n = 2) | 1.40 ± 0.51 |
| 278 (n = 1) | 1.297 | 1718 (n = 1) | 1.49 |
| 280 (n = 1) | 0.675 | 1764 (n = 4) | 0.91 ± 0.14 |
| 290 (n = 4) | 1.10 ± 0.20 | 1886 (n = 1) | 0.792 |
| 373 (n = 2) | 1.29 ± 0.35 | 1916 (n = 2) | 0.93 ± 0.24 |
| 375 (n = 4) | 1.23 ± 0.28 | 2264 (n = 1) | 0.820 |
| 378 (n = 1) | 0.862 | 2407 (n = 1) | 1.151 |
| 380 (n = 3) | 0.73 ± 0.22 | 2515 (n = 1) | 0.663 |
| 404 (n = 1) | 0.951 |  |  |

**ST: sequence type;**
